# In Silico Characterisation of the *Aedes aegypti* Gustatory Receptors

**DOI:** 10.3390/ijms241512263

**Published:** 2023-07-31

**Authors:** Maria Bibi, Adil Hussain, Farman Ali, Asad Ali, Fazal Said, Kaleem Tariq, Byung-Wook Yun

**Affiliations:** 1 Department of Entomology, Abdul Wali Khan University Mardan, Mardan 23200, Khyber Pakhtunkhwa, Pakistan; 2 Department of Applied Biosciences, College of Agriculture and Life Sciences, Kyungpook National University, Daegu 41566, Republic of Korea

**Keywords:** *Aedes aegypti*, gustatory receptors, characterization, nitric oxide, S-nitrosylation

## Abstract

*Aedes aegypti*, also known as the dengue mosquito or the yellow fewer mosquito, is the vector of dengue, chikungunya, Zika, Mayaro and yellow fever viruses. The *A. aegypti* genome contains an array of gustatory receptor (GR) proteins that are related to the recognition of taste. In this study, we performed in silico molecular characterization of all 72 *A. aegypti* GRs reported in the latest version of *A. aegypti* genome AaegL5. Phylogenetic analysis classified the receptors into three major clads. Multiple GRs were found to encode multiple transcripts. Physicochemical attributes such as the aliphatic index, hydropathicity index and isoelectric point indicated that *A. aegypti* gustatory receptors are highly stable and are tailored to perform under a variety of cellular environments. Analysis for subcellular localization indicated that all the GRs are located either in the extracellular matrix or the plasma membrane. Results also indicated that the GRs are distributed mainly on chromosomes 2 and 3, which house 22 and 49 GRs, respectively, whereas chromosome 1 houses only one GR. NCBI-CDD analysis showed the presence of a highly conserved 7tm_7 chemosensory receptor protein superfamily that includes gustatory and odorant receptors from insect species *Anopheles gambiae* and *Drosophila melanogaster*. Further, three significantly enriched ungapped motifs in the protein sequence of all 72 *A. aegypti* gustatory receptors were found. High-quality 3D models for the tertiary structures were predicted with significantly higher confidence, along with ligand-binding residues. Prediction of S-nitrosylation sites indicated the presence of target cysteines in all the GRs with close proximity to the ligand-bindings sites within the 3D structure of the receptors. In addition, two highly conserved motifs inside the GR proteins were discovered that house a tyrosine (Y) and a cysteine (C) residue which may serve as targets for NO-mediated tyrosine nitration and S-nitrosylation, respectively. This study will help devise strategies for functional genomic studies of these important receptor molecules in *A. aegypti* and other mosquito species through in vitro and in vivo studies.

## 1. Introduction

*Aedes aegypti* (also known as the Asian tiger mosquito) is a species of mosquito and a member of the family Culicidae and order Diptera. It is also known as the yellow fever mosquito. *A. aegypti* is a vector for the spread of multiple arboviruses such as yellow fever (YFV), chikungunya virus (CHIKV), Zika virus (ZIKV), Mayaro virus (MAYV) and dengue virus (DENV) and multiple other animal diseases. Factors like overpopulation, poor water draining and poor storage systems contribute to the spread of *A. aegypti*-carried diseases. *A. aegypti* breeding sites include construction sites, small and/or large water bodies and household areas in cities. Adult females need blood to produce eggs. The eggs, which are laid in water, hatch to release larvae (also known as wigglers) that appear to hang upside down in the water; the larvae go through four larval instars before pupation. Adult mosquitoes emerge from pupae and fly away. It takes about 7–10 days for an egg to develop into an adult mosquito [1]. The life span of adult *Aedes aegypti* is about 2 to 4 weeks, depending on food availability and environmental conditions [2]. There are three forms of *Aedes aegypti*, i.e., sylvan, domestic and peridomestic. The domestic form breeds and lives in urban areas. The sylvan form generally lives and breeds in forest ecosystems, whereas the peridomestic type is found in farms and groves [3].

The size of an adult *A. aegypti* is about 4–7 mm [4]. However, adult females are usually larger than males, with short hairs on their antennae. Male adults have feathery-type (plumose) antennae. Body colour is dark with scattered white spots on the body, especially on the legs, which is how they are differentiated from other mosquito species (Supplementary Figure S1A). *A. aegypti* adults have white scales on the upper surface of the thorax in the form of a lyre (Supplementary Figure S1B). Male mouthparts are adopted for nectar feeding, whereas the female proboscis is modified for blood feeding [5]. The abdomen of the female mosquito expands after blood feeding, visible with a dark red colour (Supplementary Figure S1C).

Receptor molecules are proteins in nature, and their main function is to receive and transduce the received signals [6] that originate from the perception of various external stimuli. Receptor molecules are often situated in the cell membrane. These molecules represent an amazing natural interface between chemistry and biology [7], as they convert chemical molecules received from outside the cell (e.g., odour or taste) and deliver them inside the cell in the form of electrical signals. Furthermore, receptor molecules work to either relay (send the signal onward), amplify (increase its intensity or effect) or integrate (incorporate the signal into a biomolecule) the signal. Receptor function usually involves binding to other endogenous or exogenous molecules, which are known as ligands. There are several ways to classify receptor molecules such as their location or function. Insects can detect various chemicals in their surroundings. Three basic types of insect olfactory receptors (ORs) include the ionotropic receptors (IRs), olfactory receptors (ORs) and taste-related gustatory receptors (GRs), which are the focus of this study.

Insect GRs comprise a large family of G-protein-coupled receptors responsible for the recognition of taste, although they are evolutionarily different from the taste receptors of other animals. The GR family members were first identified in *Drosophila melanogaster* mouthpart tissues [8]. In *A. aegypti*, a varying number of gustatory receptor neurons are housed within the sensilla located in the taste organs (tarsi, labellum, labrum and cibarium) of different insects such as legs, wings, abdomen and proboscis. For example, the tarsi-based trichoid sensilla have up to five gustatory neurons, the labellum- and labrum-based sensilla typically house up to four neurons, and the cibarial sensilla house up to three neurons [9,10,11,12]. These neurons co-express multiple genes of the family of gustatory receptors. The combination of gustatory receptors and olfactory receptors forms a superfamily of chemoreceptors [13]. Different mosquito species express a variable number of GR genes in their genomes. For example, the *Anopheles gambiae* genome has 90 GR genes, the *Culex quinquefasciatus* genome has 126 and the *A. aegypti* genome has 72 GR genes encoding 107 transcripts [14,15]. GR proteins exhibit a certain level of tissue specificity as some of them may be expressed in the labellum but not in the tarsi, whereas some may be expressed in the tarsi but not in the labellum [16]. Although putative mosquito taste receptors including GRs have been identified, the exact details of their function at the molecular level remain largely unknown, and their role in taste cues, taste coding and taste perception are some of the topics that remain to be explored.

The *Aedes aegypti* genome has been sequenced. It is a relatively larger genome (~1.25 Gb) distributed over its three chromosomes and is significantly repetitive. The AaegL3 genome sequence [17] provided a good resource for genome-related studies in *A. aegypti* and other mosquito species. However, it was not fully secured to a physical chromosomal map [18]. Subsequently, the AaegL4 genome assembly published in 2017 [19] also suffered from short contigs and a large number of gaps. More recently, Matthews et al. (2018) [14] published a more detailed and in-depth AaegL5 genome and AaegL5.0 annotation.

Nitric oxide plays a key role in the life of bloodsucking insects such as *A. aegypti*. Bloodsucking insects such as kissing bugs, bed bugs, ticks and mosquitoes inject NO via their saliva, which contains NO-releasing heme proteins called nitrophorins [20,21]. The NO thus released results in blood vessel dilation, thereby making it easier for the insects to suck blood. Although the molecular details of this mechanism are not well understood, it clearly indicates a highly important and strict control of the interaction between NO, parasite saliva and the taste or gustatory receptors. NO is already known to regulate a plethora of physiological processes in different species via regulation of the receptor molecules via post-translational molecules, and the same may well be applied to the insect gustatory receptors. Nitric oxide is a colourless gas. It is one of the most important oxides of nitrogen in biological systems. It has an unpaired electron on the nitrogen (^•^N=O) atom that makes it highly reactive [22]. In plants and animals including humans, NO plays a critical role as a signalling molecule in many biological processes [23]. NO plays a critical role in regulating plant physiology under normal and stress conditions [24,25,26,27,28]. In animals, NO plays a key role in the treatment of asthma and cardiovascular disease [29]. Bacterial NO prolongs the life span of nematodes [30] and promotes the jasmonate-dependent plant defence against root-knot nematodes [31]. NO is also involved in regulating insect immunity [32]. In bloodsucking insects, NO is secreted from the salivary glands and transferred to the host to act as a vasodilator or platelet-aggregation inhibitor [20]. NO is associated with neuronal processing and long-term memory of chemo- and visual signals in honeybees [33] and plays a crucial role in producing light in fireflies [34,35]. Interestingly, NO also plays a critical role in regulating highly complex social behaviours such as the decision to flee and post-conflict depression of aggression (fight-or-flight response) [36]. On the other hand, NO fumigation has been used to control pest insects and increase post-harvest shelf life and the quality of fruits and vegetables [37]. The production of NO has been a hot topic in plant sciences. As such there are several routes for NO production in plants (reviewed by Hussain et al., 2022 [38]). In animals, it is produced by the action of the nitric oxide synthase enzyme (NOS). NO exerts its function via post-translational modifications (PTMs), of which S-nitrosylation is probably the most important and widely studied PTM. The covalent attachment of NO to the thiol group of a solvent-exposed cysteine molecule is known as S-nitrosylation (–SNO) [39]. Similarly, the attachment of NO to a tyrosine residue is known as tyrosine nitration. These PTMs have a significant impact on protein structure, function and interaction. PTMs represent a natural mechanism in place to control protein function and accommodate an unlimited number of functions through a rather limited set of genes. In insects, the NOS enzyme has been identified in honeybee (*Apis mellifera*), fruit fly (*Drosophila melanogaster*) and locust (*Schistocerca gregaria*) [40] and has similar chemical properties to that of the vertebrate NOS [41]. There are several published reports describing the significant impact of NO-mediated PTMs on the function of different receptor molecules in different species. The objective of this study is to characterise the *A. aegypti* gustatory receptors and try to predict how NO might affect the function of these receptor molecules via putative post-translational modification.

## 2. Results

### 2.1. Identification of A. aegypti Gustatory Receptors and Phylogenetic Analysis

**Figure 1:**
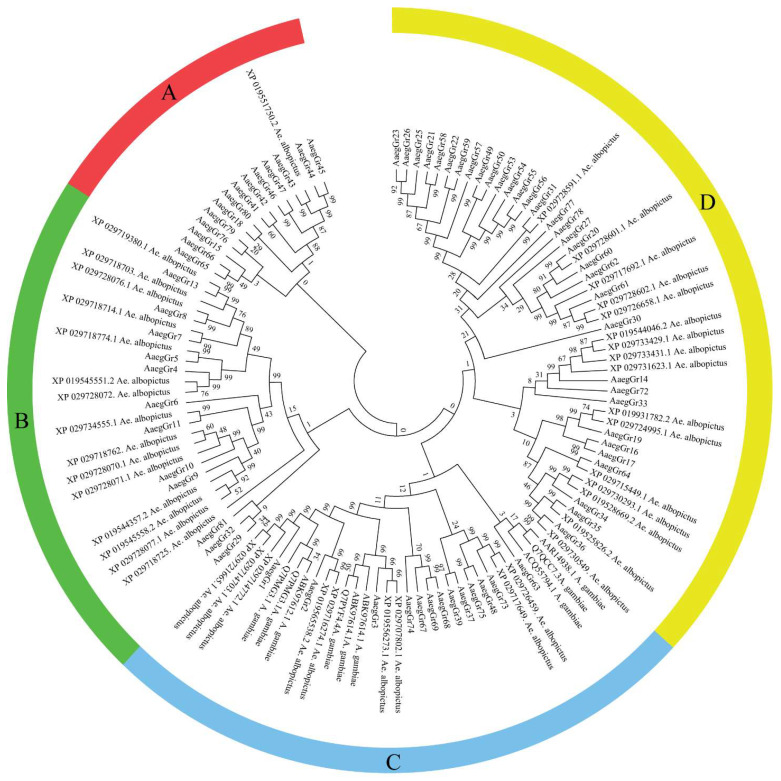
Evolutionary relationships of *A. aegypti* gustatory receptors. The evolutionary history was inferred using the neighbour-joining method [42]. The bootstrap consensus tree inferred from 1000 replicates [43] is taken to represent the evolutionary history of the taxa analysed [2]. Branches corresponding to partitions reproduced in less than 50% of bootstrap replicates are collapsed. The evolutionary distances were computed using the Poisson correction method [44] and are in the units of the number of amino acid substitutions per site. The analysis involved 122 amino acid sequences. All ambiguous positions were removed for each sequence pair. There were a total of 611 positions in the final dataset. Evolutionary analyses were conducted in MEGA7 [45].

A search of various databases provided a variable number of gustatory receptor genes. We found a total of 72 gustatory receptors in the AaegL5 genome which encoded a total of 107 transcripts. Among these, eight genes encoded multiple transcripts with GR19 encoding 3 transcripts, GR20 encoding 13, GR33 encoding 2, GR39 encoding 8, GR60 encoding 4, GR61 encoding 3 and GR67 and GR74 encoding 5 transcripts each of varying sizes. Furthermore, we also found 9 GR protein sequences for *Anopheles gambiae* and 41 for *Ae. albopictus*. Phylogenetic analysis divided the receptors into four major clads: A, B, C and D (Figure 1). Clad A was the smallest clad with 14 AaegGRs and only 1 GR from *Ae. albopictus*. Clad B contained 12 AaegGRs and 15 GRs from *Ae. albopictus*. Clad C contained a blend of 31 GRs from *Ae. albopictus* (9), *Anopheles gambiae* (9) and *A. aegypti* (13). Clad D, which was the largest clad, contained a total of 49 gustatory receptors with 33 AaegGRs and 16 GRs from *Ae. albopictus* but none from *Anopheles gambiae* (Figure 1).

### 2.2. Physiochemical Characterisation and Chromosomal Distribution of A. aegypti Gustatory Receptors

**Figure 2:**
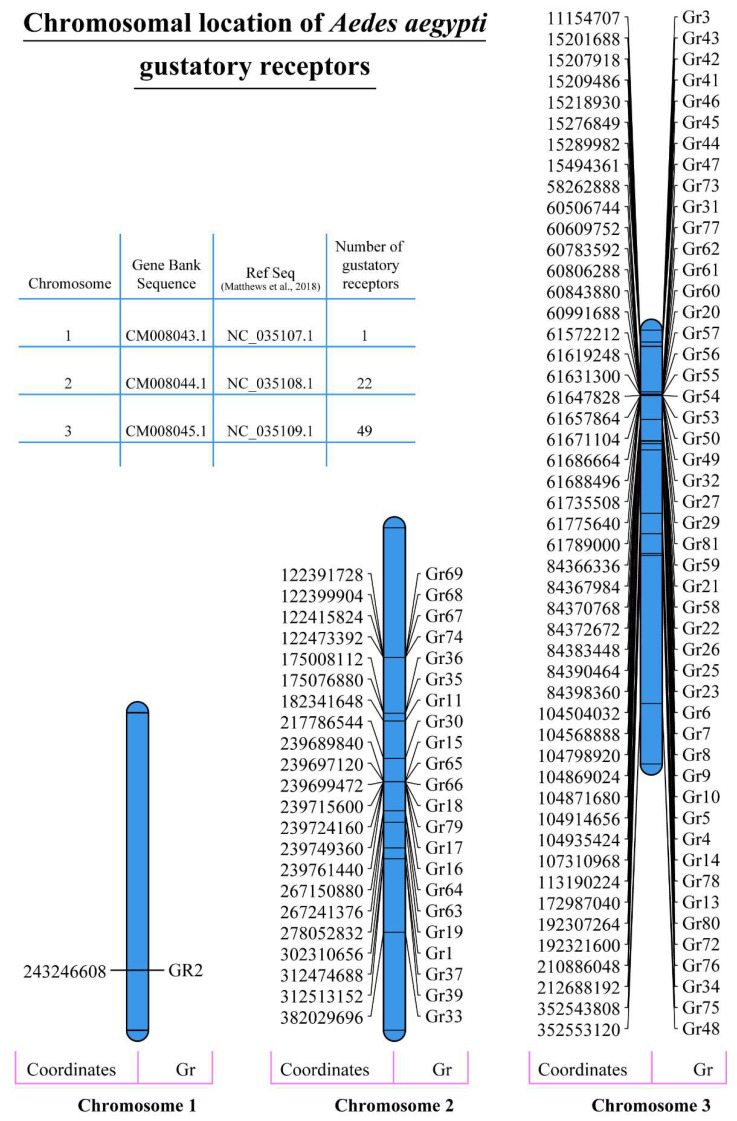
Chromosomal distribution of *A. aegypti* gustatory receptors. Data on the chromosomal distribution of *A. aegypti* gustatory receptors and gene chromosomal coordinates were obtained from different resources such as the Gene Bank and Matthews et al. (2018) [14]. The data were used to graphically represent the chromosomal location of all the GR genes on their respective chromosomes drawn to size using MapChart [49].

The physical and chemical properties of proteins are extremely important in determining their function. Our analysis revealed that *A. aegypti* gustatory receptors are relatively larger in size as reflected by their higher molecular weight. A total of 19 GR proteins showed a molecular weight of more than 50 kda, 52 GR proteins had a molecular weight of more than 40 kda and just one had a molecular weight of 35.2 kda (Table 1). According to Guruprasad et al. (1990), the instability index provides an estimation of the stability of a protein in a test tube [46]. Proteins with an instability index of less than 40 are considered stable proteins [46]. A total of 48 GR proteins showed an instability index of less than 40, whereas the remaining proteins had instability index values between 40 and 50 (Table 1). Furthermore, A. Ikai (1980) correlated the thermostability of a protein with its aliphatic index [47], which may be defined as the relative volume of the aliphatic side chains (leucine, isoleucine, valine and alanine) and represents the thermostability of proteins. Generally speaking, aliphatic amino acids are also hydrophobic in nature. Interestingly, 64 GR proteins showed an aliphatic index of more than 100, whereas the remaining 8 GR proteins had an aliphatic index between 95 and 99 (Table 1). The hydrophobic or hydrophilic nature of a protein plays an extremely important role in its function within the cellular environment. A hydropathicity index is a number indicating the hydrophobic or hydrophilic properties of a protein [48]. The values for this number usually range between 4.5 (for the most hydrophobic amino acid isoleucine) and −3.9 (for the most hydrophilic amino acid lysine). The hydropathic values for all the gustatory receptor proteins analysed in this study ranged between 0.1 and 0.7 (Table 1). This indicates the slight hydrophobic nature of these proteins. The isoelectric point (pI) value shows the pH at which a protein molecule is electrically neutral. A total of 30 gustatory receptors showed a pI value above 9, 36 receptors had a pI value between 7 and 9 and only 3 GRs had a pI value between 5 and 7. (Table 1). Analysis conducted to predict the cellular localization of *A. aegypti* gustatory receptors via WoLFPSORT found localization signals from several cellular locations. However, we chose to show only those subcellular locations for which the analysis found more than five known identical genes. Results showed that all the gustatory receptors are either expressed in the extracellular matrix or the plasma membrane, which is typically expected for receptor proteins (Table 1). Analysis for chromosomal distribution of gustatory receptors showed that *A. aegypti* chromosome 1 houses only 1 GR (GR2), whereas chromosomes 2 and 3 express 22 and 49 GRs, respectively (Figure 2). The same was reported earlier by Matthews et al. [14].

### 2.3. Identification of Conserved Domains and Motifs

**Figure 3:**
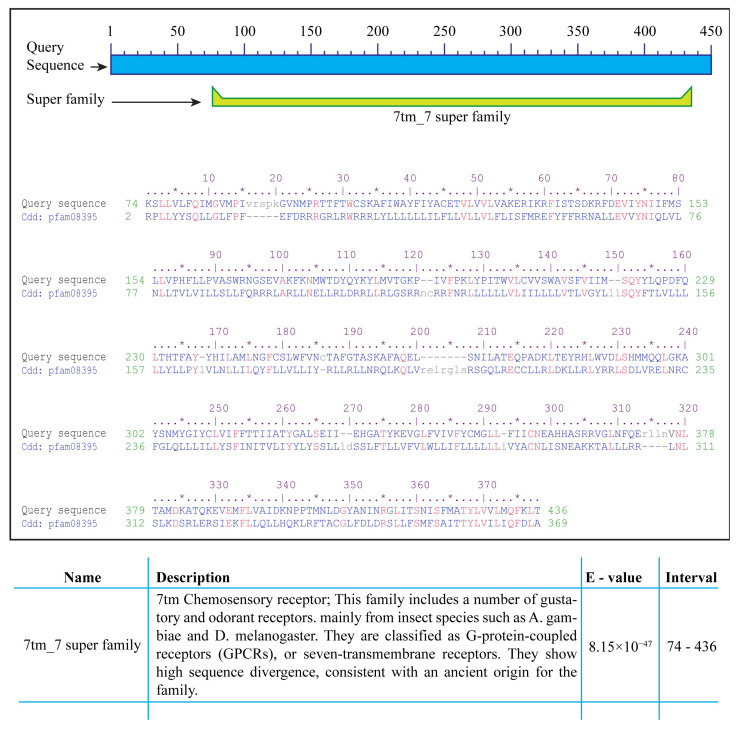
Conserved domains in *A. aegypti* gustatory receptors. Analysis via NCBI-CDD indicated the presence of a highly conserved 7tm_7 superfamily domain of approximately 360 amino acids in all 72 *A. aegypti* gustatory receptors. Proteins containing the pfam7tm_7 domain are a Pfam family of G-protein-coupled receptors (GPCRs) or seven-transmembrane (7tm) chemosensory receptors including gustatory and odorant receptors from insects such as *Anopheles gambiae* and *Drosophila melanogaster*. * indicates fifth amino acid to make the counting easy. Different amino acids are colored blue, similar amion acids are colored red whereas, unique amino acids are coloured grey and written in small caps.

Analysis for the presence of conserved domains via NCBI-CDD indicated the presence of a ~360 amino acid 7tm_7 superfamily domain in the protein sequence of all 72 gustatory receptors. According to Pfam database annotation, the domain with Pfam ID 7tm_7 is present in the 7tm chemosensory receptor proteins, which is a protein family including gustatory and odorant receptors from insect species *Anopheles gambiae* and *Drosophila melanogaster*. The proteins are classified as G-protein-coupled receptors (GPCRs) or seven-transmembrane (7tm) receptors (Figure 3) [13,50].

**Figure 4:**
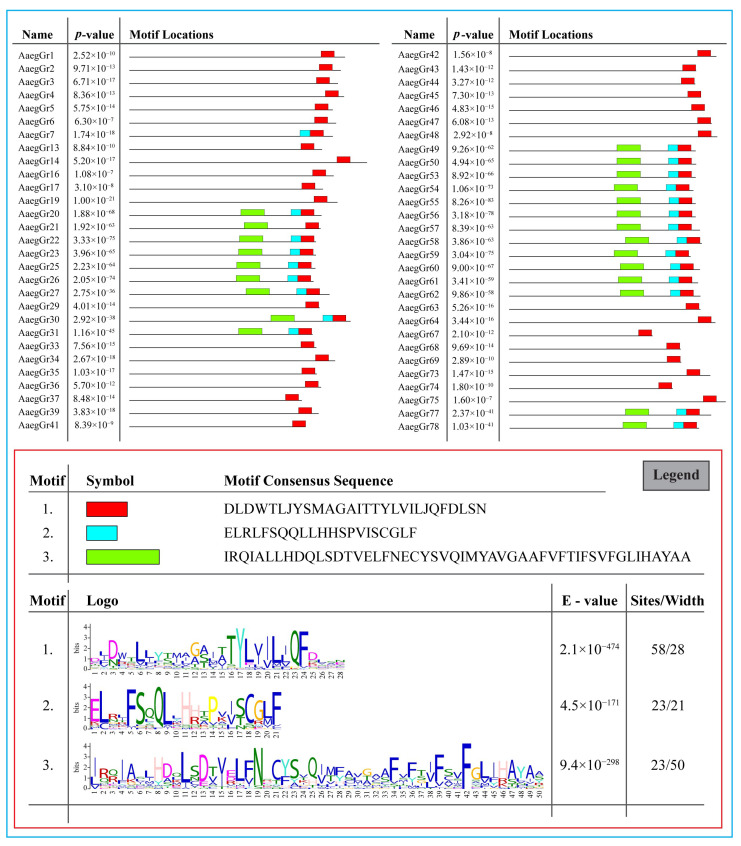
Identification of conserved motifs via MEME (Bailey and Elkan 1994—[51]). Three significantly enriched ungapped motifs were found in the protein sequence of all 72 *A. aegypti* gustatory receptors. The number of sites contributing to the construction of the discovered motifs was 58, 23 and 23 for motifs 1, 2 and 3, respectively, with a width of 28, 21 and 50 amino acids, respectively.

Furthermore, we found at least three significantly enriched ungapped motifs in the protein sequence of all 72 *A. aegypti* gustatory receptors. The motifs were located more towards the end of the protein sequences. The number of sites contributing to the construction of the discovered motifs was 58, 23 and 23 for motifs 1, 2 and 3, respectively, with a width of 28, 21 and 50 amino acids, respectively (Figure 4 legend). The consensus sequences for the motifs are shown in Figure 4 below.

### 2.4. Homology Modelling and Prediction of S-Nitrosylation Sites in GR Proteins

**Figure 5:**
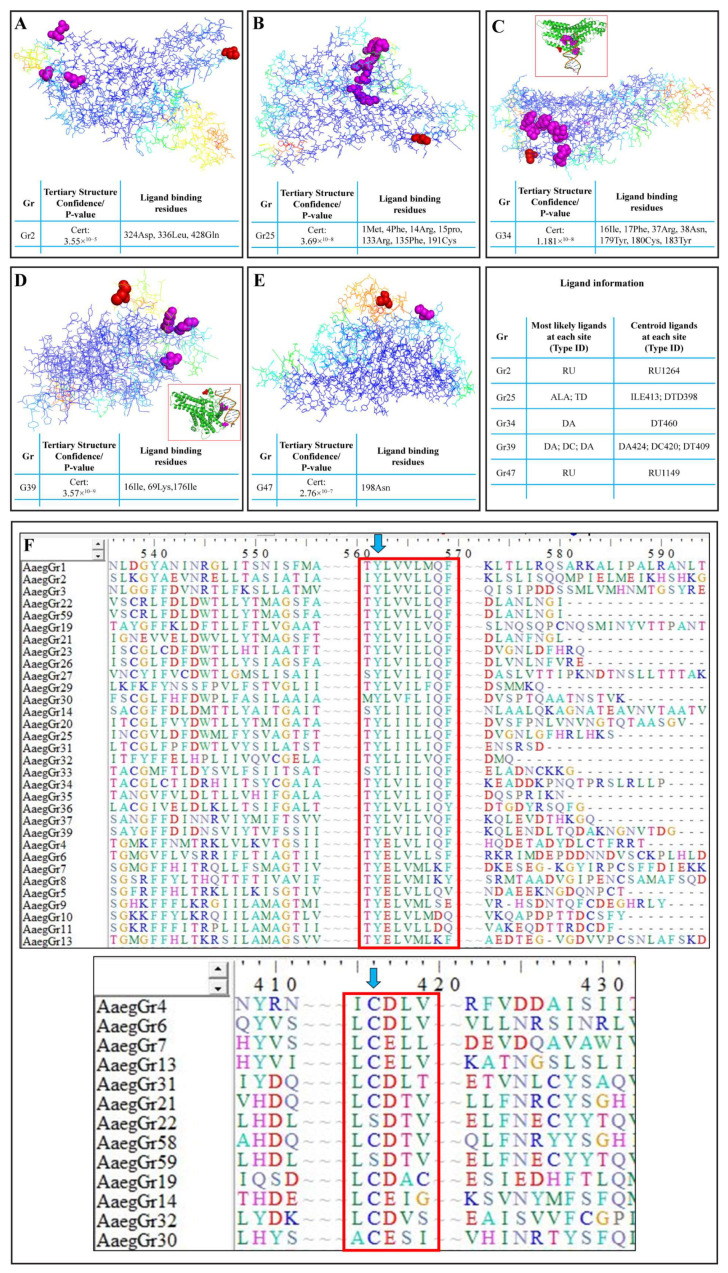
Homology modelling and prediction of S-nitrosylation sites in *A. aegypti* gustatory receptors: The predicted 3D tertiary structure of (**A**) GR2; (**B**) GR25; (**C**) GR34; (**D**) GR39; (**E**) GR47. (**F**) The identification of highly conserved motifs in GR proteins and targets of NO-mediated tyrosine nitration and S-nitrosylation. Tables indicate the confidence/*p*-value of the tertiary structure prediction and the potential ligand-binding residues. Magenta spheres in the tertiary structure indicate the ligand-binding residues, whereas the red spheres indicate potential S-nitrosylation targets. The tertiary structures shown here use the reverse rainbow colour scheme with blue (high accuracy) to green, and yellow and orange to red (low accuracy).

The 3D models of the tertiary structures of GR proteins were predicted using SWISS-MODEL. Only the most significant structures with the lowest *p*-values were selected for further analysis in Pymol. Results indicated a significantly higher accuracy of the tertiary structure shown by the reverse rainbow colour scheme with blue (high accuracy) to green, and yellow and orange to red (low accuracy) (Figure 5). Homology models were downloaded for all the GR proteins and indicated significantly higher structural characteristics. In Figure 5, we show the structures of a few GR proteins which were also predicted to be the potential targets for S-nitrosylation (Table 2) and which also had potential ligands with predicted binding sites. For example, the GR2 tertiary structure was predicted with a *p*-value of 3.55 × 10^−5^. It was also predicted that this receptor binds to a potential ligand at the amino acid residues 324Asp, 335Leu and 428Gln (Figure 5A—magenta spheres). Furthermore, GR2 was also found to be a potential target for NO-mediated S-nitrosylation at Cys270 (Table 2 and Figure 5A—red spheres). Similarly, seven amino acids were identified as ligand-binding sites in GR25 (Figure 5B—magenta spheres) with Cys343 as a potential target of S-nitrosylation (Figure 5B—red spheres). GR34 structural and ligand-binding prediction indicated seven different amino acids as potential DNA-binding sites (Figure 5C—magenta spheres and inset) with Cys51 and Cys368 as potential S-nitrosylation targets (Table 2 and Figure 5C—red spheres). Similarly, GR39 showed three amino acids (16Ile, 69Lys and 176Ile) as potential DNA-binding sites (Figure 5D—magenta spheres and inset). In addition, GR39 also housed Cys7 as an S-nitrosylation target (Table 2 and Figure 5D—red spheres). GR47 was another receptor protein found to house the S-nitrosylation target Cys12 (Table 2 and Figure 5E—red spheres) as well as the potential ligand-binding site 198Asn (Figure 5E—magenta spheres). Some of the gustatory receptors were found to have multiple cysteine residues as potential S-nitrosylation targets. For example, GR14, GR34, GR43, GR47, GR60, GR62 and GR73 housed two cysteines each, whereas GR76 contained five cysteine residues as potential S-nitrosylation targets (Table 2).

Furthermore, during the phylogenetic analyses, we observed that at least two motifs in the amino acid sequences of more than 32 of the receptors were highly conserved. The first motif, TYL/EV/LV/IL/MM/LQF (Figure 5F—top red box), was situated either at the middle or end of the amino acid sequence. Similarly, another upstream motif found in 13 receptors was the I/LCD/EL/TV motif (Figure 5F—bottom red box). These two motifs appear to play a key role in the function of these receptors in part because they are highly conserved and in part because of another significantly special feature, i.e., the first motif houses a tyrosine residue (Y), whereas the second motif houses a cysteine (C) residue (Figure 5F—blue arrows), which are the target sites for NO-mediated tyrosine nitration and S-nitrosylation, respectively.

## 3. Discussion

*Aedes aegypti*. L. (Diptera: Culicidae) is an important mosquito species responsible for the spread of several diseases in animals including humans. *A. aegypti*-transmitted dengue fever is of particular importance in underdeveloped and developing countries like Pakistan where it is a nationwide risk. In their final dengue response report for Pakistan, the International Federation of Red Cross and Red Crescent Societies reported 22,938 cases in 2017, and 3200, 24,547 and 3442 cases in 2018, 2019 and 2020, respectively. A significant increase in reported cases was observed in 2021 as 48,906 cases were reported (January to November) including 183 deaths with a case fatality ratio (CFR) of 0.4%. Reported cases were particularly higher in the developed and highly populated cities including the capital city Islamabad (www.ifrc.org, accessed on 10 June 2022). This resulted in nationwide health emergency responses incurring huge monetary expenses. This indicates the extremely high success of *A. aegypti* as a mosquito species in Pakistan. This is in part because of the deteriorating health and sanitary conditions of populated cities.

However, the success of *A. aegypti* is also because of its highly developed chemoreception capabilities. Chemoreceptors present an interface between the chemical world and biology. Of these, the taste receptors are largely understudied and play an extremely important role in host selection, mating and feeding responses. An improved understanding of the mosquito taste receptors will provide new insights into several aspects of their behaviour. Keeping this in view, we characterised the gustatory receptor armada of *A. aegypti*. For this, we used the DNA and amino acid sequences of the 72 gustatory receptors from the latest version of *A. aegypti* genome AaegL5 [14].

We compared the proteins sequences of GRs from *A. aegypti*, *A. albopictus* and *A. gambiae*. Our analysis indicated a significantly closer evolutionary relationship between *A. aegypti* and *Ae. albopictus* than *A. gambiae*, which is understandable keeping in mind that the latter is a completely separate genus now. However, the number of *A. gambiae* GRs was rather limited as compared to the other two mosquito species. The physicochemical attributes determine the function of proteins. As expected, the *A. aegypti* gustatory receptors were found to be relatively larger in size as reflected by their higher molecular weights (>50 kda). Per the definition of Guruprasad et al. (1990) [46], GR proteins were found to be generally stable as reflected by their instability index of less than 40. The thermostability of 64 GR proteins was further supported by the aliphatic index of more than 100, indicating the hydrophobic nature of these proteins [47] which is further supported by the hydropathicity index values. Generally speaking, the hydrophobic amino acids tend to be inside the three-dimensional shape of a protein, whereas the hydrophilic amino acids are found toward the outer surface. Together, the significantly higher aliphatic index and slight hydrophobicity make the *A. aegypti* gustatory receptor proteins highly stable under a variety of environmental conditions. The relatively higher isoelectric points of most GRs indicate that these receptors are tailored to perform under high pH or basic cellular environments. It is, however, important to mention that protein folding, post-translational modifications and labelling can influence and modify the pI value. Our results also showed that all the gustatory receptors are either expressed in the extracellular matrix or the plasma membrane, which is typically expected for receptor proteins as they function at the interface between the cellular and outside environments by receiving signals from outside the cell and communicating them downstream inside the cell.

Analysis for chromosomal distribution of gustatory receptors showed that *A. aegypti* chromosome 1 houses only 1 GR, whereas chromosomes 2 and 3 express 22 and 49 receptors, respectively. According to genome biologists, the chromosomal location of genes plays a key role in the variation and evolution of various traits in living organisms. Such traits may differ among individuals but vary significantly across populations. As such, chromosomes hold a lot of genes, some in the middle and others at the end of their linear structures. Genes located in the middle have been found to contribute less to genetic variations over time as compared to the genes found at the ends of a chromosome [52]. Furthermore, the chromosomal location of genes from the same family plays a key role in their function and functional redundancy. The chromosomal location is also important to determine whether homologous genes are recently duplicated pseudogenes or are splice variants of the same gene. This is particularly important in the case of *A. aegypti* gustatory receptors, as Matthews et al. [14] reported 12 different pseudogenes in the latest version of the *A. aegypti* genome (AaegL5). Furthermore, according to Campbell et al. [53], sex determination in *A. aegypti* is controlled by a dominant male-determining locus on chromosome 1. Male mosquitoes are heterozygous (Mm) for this locus. There are no sex chromosomes in *A. aegypti*, and sex determination alleles have been linked to the smallest homomorphic autosome 1. Since only female mosquitoes feed on blood, the presence of only one GR on the sex-determining chromosome 1 is certainly interesting. However, as sex is controlled by a dominant male-determining locus, the presence or absence of this locus determines the sex of an individual mosquito, but the distribution of other genes such as the GRs on chromosomes 1, 2 and 3 will not directly affect sex determination and their inheritance should follow standard Mendelian inheritance patterns. Since Chr1, 2 and 3 are all autosomes, we assume that the GRs located on them will be inherited in a similar manner in both males and females. However, specific inheritance patterns of alleles would depend on their dominant or recessive nature and whether the parents are homozygous or heterozygous.

Signals received by the receptors are usually transmitted downstream via physical binding and attachment to other proteins known as ligands. The three-dimensional structure and chemical properties of the protein play a key role in determining the binding sites and type of ligands. We found ligand-binding sites for several GR proteins. However, we also found cysteine residues as significant potential targets of NO-mediated S-nitrosylation. Interestingly, these ligand-binding sites and the S-nitrosylation targets were found to be significantly closer to each other within the tertiary structure. This indicates a higher probability of a significant impact of S-nitrosylation events on the ligand-binding activity of the gustatory receptors as S-nitrosylation may completely change the chemical nature and folding of the protein. This phenomenon can have significant biological consequence including variable or preferential stimuli perception or preferential response to taste, smell or human blood or even hunger. Further investigations in this direction will provide valuable insights about *A. aegypti* behaviour, host detection and preference and feeding behaviour.

Furthermore, we identified two highly conserved motifs inside GR proteins. These motifs house a tyrosine (Y) and a cysteine (C) residue, both the targets of NO-mediated tyrosine nitration and S-nitrosylation, respectively, indicating the presence of a molecular switch for mediating the function of these key receptor molecules. Several studies have reported NO-mediated PTMs modulating the function of key receptor proteins in other species such as the S-nitrosylation of auxin receptor TIR1 in plants [54,55], S-nitrosylation of ABI5 involved in abscisic acid signalling in plants [56], S-nitrosylation of NPR1, the master regulator of salicylic-acid-mediated plant defence [57] and S-nitrosylation of JAZ1 involved in jasmonic-acid-dependent plant defence [58].

Similarly, S-nitrosylation of key proteins from the class Insecta has also been reported. For example, Weichsel et al. (2005) [59] reported that the NO storage and delivery protein nitrophorin is reversibly S-nitrosylated at its proximal heme cysteine (Cys60) in the bloodsucking insect *Cimex lectularis* (bedbug). Furthermore, NO also directly binds to the heme centre of this protein, making it an important cellular reservoir of NO in this bloodsucking insect. This indicates that NO-mediated S-nitrosylation plays an important role in regulating the function of key proteins. This study provides the first molecular characterisation of the *A. aegypti* gustatory receptor proteins. The findings of this study will help devise strategies for functional genomic studies of these important receptor molecules in *A. aegypti* and other mosquito species. Further evaluation and validation of these findings through in vitro and in vivo studies are recommended for prospective researchers.

## 4. Materials and Methods

### 4.1. Identification of A. aegypti Gustatory Receptors and Phylogenetic Analysis

The *Aedes aegypti* receptor genes were identified by searching the latest version of *A. aegypti* genome AaegL5 [14] on the Ensemble platform Ensembl Metazoa (http://metazoa.ensembl.org/Aedes_aegypti_lvpagwg/Info/Index, accessed on 10 June 2022) of the European Bioinformatics Institute, European Molecular Biology Laboratory (EMBL-EBI, Cambridge, UK). The protein sequences of the 72 identified gustatory receptors were downloaded for further analysis. Furthermore, 41 GR protein sequences for *Ae. albopictus* and 9 for *Anopheles gambiae* were also downloaded from NCBI. To identify the similarities, dissimilarities and evolutionary relationships among the identified GRs, phylogenetic analysis was performed through MEGA 7 (https://www.megasoftware.net/, accessed on 10 June 2022) [60]. For phylogenetic analysis, the protein sequences of all 122 gustatory receptors were imported into MEGA 7 to perform multiple sequence alignment (MSA) using CLUSTALW with default settings. The MSA was used to generate a phylogenetic tree by using the neighbour-joining method with 1000 bootstraps, with the Poisson correction and pair-wise deletion method.

### 4.2. Physiochemical Characterisation

The physiochemical and molecular characterisation of *A. aegypti* gustatory receptors was performed by using the Swiss Bioinformatics Resource Portal Expasy-ProtParam (https://www.expasy.org/resources/protparam, accessed on 10 June 2022). Physicochemical attributes such as the isoelectric point (pi), molecular weight, instability index, hydrophilic or hydrophobic nature and other attributes of the GR proteins were determined. Moreover, the subcellular localization of the gustatory receptor protein was predicted by using WoLF PSORT (https://wolfpsort.hgc.jp/, accessed on 10 June 2022) NAKAI Lab, Tokyo, Japan.

### 4.3. Chromosomal Distribution of A. aegypti Gustatory Receptors

Data on the chromosomal distribution of *A. aegypti* gustatory receptors were obtained from different genome resources, mentioned above. Genomic coordinates for the transcriptional start and stop sites were searched. The data were used to graphically represent the chromosomal location of all of the GR genes on their respective chromosomes drawn to size using MapChart (https://www.wur.nl/en/show/mapchart.htm, accessed on 10 June 2022) software by Roeland E. Voorrips in 2002 at Wageningen University [49]. Data from MapChart were saved as an enhanced metafile (EMF) and further processed for colouring and labelling.

### 4.4. Identification of Conserved Domains in A. aegypti Gustatory Receptors

The amino acid sequence of GR genes was used to identify conserved domains on the conserved domain database (CDD) of the NCBI (https://www.ncbi.nlm.nih.gov/Structure/bwrpsb/bwrpsb.cgi, accessed on 10 June 2022). Conserved motifs were also identified via multiple sequence alignment using BioEdit version 7.0 [61].

### 4.5. Motif Composition Analysis

Multiple Em for Motif Elicitation (MEME) (https://meme-suite.org/meme/tools/meme, accessed on 10 June 2022) [51] was used to identify conserved motifs in the GR genes using default parameters. The location and pattern of different motifs along with their statistical *p*-value were downloaded as SVG files. Furthermore, the motif consensus sequences and logos were also downloaded as SVG files, and their statistical E-values were recorded.

### 4.6. Homology Modelling, Tertiary Structure and Prediction of Ligand-Binding and S-Nitrosylation Sites in GR Proteins

The 3D models of all the GR proteins were made using SWISS-MODEL (http://swissmodel.expasy.org, accessed on 10 June 2022) [62]. Tertiary structure predictions were performed using IntFold on the University of Reading Bioinformatics Web Servers (https://www.reading.ac.uk/bioinf/IntFOLD/, accessed on 10 June 2022) [63,64]. The top-most tertiary structure with the highest confidence and lowest *p*-value were downloaded as PDB files and displayed via Pymol (https://pymol.org/2/, accessed on 10 June 2022). Furthermore, ligand-binding-site prediction was performed via FunFold on the University of Reading Bioinformatics Web Servers [65]. Data on the location of ligand-binding sites were recorded, and 3D structure files were downloaded as PDF files. To identify potential candidates for S-nitrosylated GRs, full-length protein sequences were analysed via GPS-SNO (http://sno.biocuckoo.org/, accessed on 10 June 2022) [66] with a high threshold. Data were recorded in the form of a table, and the target cysteines were shown as red spheres in the predicted 3D structure of the respective proteins.

## Figures and Tables

**Table 1:** Physicochemical attributes of *A. aegypti* gustatory receptor proteins.

Receptors	Physicochemical Properties						Subcellular Localization	
Gustatory Receptor	M Weight (da)	Instability Index	Aliphatic Index	Hydropathicity (GRAVY)	Length	pI	Extracellular Matrix	Plasma Membrane
AaegGr1	53,444.62	41.52	98.35	0.101	460	9.04	18	12
AaegGr2	51,563.44	32.05	113.08	0.394	451	6.63	20	10
AaegGr3	50,713.64	41.93	101.98	0.411	445	8.28	23	7
AaegGr4	53,284.49	36.36	95.94	0.17	458	9.59	16	11
AaegGr5	50,232.77	35.02	101.01	0.225	434	9.43	19	8
AaegGr6	51,286.81	50.57	104.29	0.254	441	8.93	18	9
AaegGr7	50,049.73	34.02	109.84	0.301	434	8.81	18	12
AaegGr8	48,289.27	41.6	99.13	0.392	412	8.74	12	10
AaegGr9	50,837.81	42.43	105.76	0.312	434	9.16	20	10
AaegGr10	53,308.31	37.88	97.93	0.274	455	9.16	18	10
AaegGr11	54,851.61	30.85	105.37	0.395	471	9.18	21	9
AaegGr13	47,425.52	41.39	109.05	0.387	412	7.6	19	9
AaegGr14	57,732.89	40	110.53	0.351	507	7.21	19	9
AaegGr15	45,520.29	35.96	112.76	0.458	381	8.61	17	7
AaegGr16	51,633.77	38.72	106.58	0.324	436	8.86	19	11
AaegGr17	48,875.03	33.83	107.8	0.3	413	9.44	20	10
AaegGr18	43,743.17	39.96	120.45	0.488	374	9.1	17	10
AaegGr19	50,495.17	40.54	110.43	0.446	444	8.53	19	10
AaegGr20	47,285.68	39.29	109.22	0.547	410	8.32	7	11
AaegGr21	47,288.6	31.81	114.14	0.395	408	8.8	22	8
AaegGr22	45,861.16	29.16	101.41	0.279	398	7.69	20	10
AaegGr23	46,417.3	37.96	106.26	0.356	398	9.08	21	9
AaegGr25	45,282.08	48.52	114.33	0.475	397	8.51	18	11
AaegGr26	45,332.87	32.87	103.94	0.316	393	9.1	22	8
AaegGr27	48,633.48	38.66	111.41	0.451	427	9.4	20	10
AaegGr29	47,496.89	40.28	107.22	0.291	406	9.17	16	11
AaegGr30	54,471.95	46.85	112.33	0.393	472	9.14	20	10
AaegGr31	44,257.42	33.02	124.4	0.732	391	6.43	22	8
AaegGr32	46,508.67	43.07	125.2	0.481	404	6.15	19	11
AaegGr33	45,832.22	43.61	125.06	0.441	399	8.63	22	8
AaegGr34	49,575.45	37.58	110.3	0.351	439	9.11	14	13
AaegGr35	46,145.76	35.65	119.1	0.467	400	9.51	22	8
AaegGr36	47,530.12	28.35	116.19	0.452	409	8.27	19	9
AaegGr37	43,274.2	43.18	114.05	0.476	368	8.61	19	9
AaegGr39	46,630.56	32.32	109.95	0.449	404	9.34	19	10
AaegGr41	44,001.3	25.44	121.25	0.705	376	8.5	19	8
AaegGr42	51,572.17	39.14	112.76	0.459	442	9.33	18	9
AaegGr43	46,364.08	36.59	121.13	0.607	399	8.78	19	8
AaegGr44	45,955.25	24.94	118.04	0.472	397	8.43	6	11
AaegGr45	47,807.09	42.35	98.66	0.253	410	6.18	19	10
AaegGr46	48,429.06	26.81	109.4	0.323	417	9.5	21	9
AaegGr47	50,471.36	32.11	111.2	0.263	432	9.43	20	10
AaegGr48	51,570.11	25.41	111.08	0.436	444	9.28	18	9
AaegGr49	46,189.63	36.04	101.61	0.273	398	9.3	8	11
AaegGr50	46,375.8	36.45	97.39	0.299	399	8.9	18	9
AaegGr53	45,703.47	37.67	112.86	0.573	398	9.29	15	12
AaegGr54	45,563.19	42.34	97.25	0.344	393	8.97	12	11
AaegGr55	46,453.84	41.32	96.26	0.352	398	9.25	19	8
AaegGr56	46,242.62	43.95	103.39	0.432	398	9.06	17	10
AaegGr57	46,338.85	39.62	117.59	0.531	407	8.74	23	7
AaegGr58	47,868.28	32.1	111.41	0.334	411	8.83	22	8
AaegGr59	44,534.76	29.95	101.27	0.251	387	7.72	20	10
AaegGr60	46,826.52	33.15	111.99	0.578	407	8.66	19	9
AaegGr61	45,627.76	28.62	116	0.599	403	7.49	6	11
AaegGr62	46,596.22	37.12	118.85	0.512	408	8.97	19	9
AaegGr63	46,553.49	47.49	116.89	0.314	408	9.24	16	9
AaegGr64	50,213.48	33.39	110.95	0.184	440	8.59	15	8
AaegGr65	46,745.81	46.41	107.04	0.371	395	8.83	20	10
AaegGr66	45,292.62	40.56	120.2	0.372	380	9.36	19	9
AaegGr67	35,207.96	31.8	121.37	0.495	306	9.33	19	9
AaegGr68	42,279.62	33.99	124.13	0.606	366	5.54	17	10
AaegGr69	42,413.22	35.1	114.47	0.597	367	7.58	21	9
AaegGr72	44,179.17	40.55	109.5	0.371	381	8.62	22	8
AaegGr73	48,550.67	33.11	100.91	0.321	429	8.74	22	8
AaegGr74	41,027.28	42.18	102.26	0.282	349	9.16	19	11
AaegGr75	53,206.08	28.21	113.92	0.496	462	9.36	19	10
AaegGr76	45,984.17	36.07	117.47	0.573	395	5.88	19	9
AaegGr77	49,365.32	45.77	114.48	0.559	431	7.13	11	10
AaegGr78	47,010.94	28.17	107.08	0.296	404	8.67	16	10
AaegGr79	46,939.44	36.47	125.07	0.417	406	8.86	20	8
AaegGr80	43,910.87	34.06	124.89	0.438	380	8.67	18	9
AaegGr81	44,339.37	32.21	104.97	0.338	390	9.96	19	11

**Table 2:** List of *A. aegypti* gustatory receptors with predicted cysteine(s) as potential targets for S-nitrosylation.

GR	Position	Peptide	Score	Cutoff	Cluster
AaegGr2	270	RKDVAIECTAAMISQ	4.842	2.443	Cluster B
AaegGr3	206	ILLPVLSCLAVIITH	3.478	2.443	Cluster B
AaegGr4	371	RTLAVSMCTAAVNDE	2.815	2.443	Cluster B
AaegGr7	158	ALLLGLACCEHLLAT	2.658	2.443	Cluster B
AaegGr8	235	FWRIEVACNGTVLPT	3.408	2.443	Cluster B
AaegGr9	8	MQAPNQHCLAQLRKW	3.147	2.443	Cluster B
AaegGr10	326	GHLILLSCANDMYFI	2.63	2.443	Cluster B
AaegGr11	285	YIDVFIICVSLVLQR	2.804	2.443	Cluster B
AaegGr13	404	GVGDVVPCSNLAFSK	3.25	2.443	Cluster B
AaegGr14	95	YMEPLMMCIDMLAAM	2.946	2.443	Cluster B
	111	NQKRLIECVERLDKV	3.163	2.443	Cluster B
AaegGr15	6	MAKISCLYRHVLK	2.668	2.443	Cluster B
AaegGr16	84	ILLTLSVCSAEILIA	3.582	2.443	Cluster B
AaegGr20	337	LVHKAINCASSSAVI	3.168	2.443	Cluster B
AaegGr23	116	ILANINDCDRKLGKL	2.652	2.443	Cluster B
AaegGr25	343	RVLKELRCFSQQLQH	2.5	2.443	Cluster B
AaegGr26	145	LSTGVWMCFSVIITL	2.495	2.443	Cluster B
AaegGr32	221	RLQLLNRCLEEMLLE	2.793	2.443	Cluster B
AaegGr33	396	QFELADNCKKG	1.738	1.484	Cluster A
AaegGr34	51	YGLGIVFCLAGLTYK	3.299	2.443	Cluster B
	368	VCNLMRTCKDSLTKE	4.049	2.443	Cluster B
AaegGr39	7	MLSFRPCRNKYIQQ	3.337	2.443	Cluster B
AaegGr43	5	MHTTCRTVFRLK	4.874	1.484	Cluster A
	345	FYNDAGRCVEQSIEM	3.424	2.443	Cluster B
AaegGr46	4	MFHCSQNPLLS	3.033	2.443	Cluster B
AaegGr47	12	KTSHKKACIHDKTYQ	1.743	1.484	Cluster A
	313	IMGVFIACVTTVNDI	2.696	2.443	Cluster B
AaegGr49	54	LVMGVFMCVGAMYYS	2.663	2.443	Cluster B
AaegGr53	251	VVVLFNKCFSKLVMF	3.495	2.443	Cluster B
AaegGr54	184	AFSWVMGCYQTLAST	2.685	2.443	Cluster B
AaegGr55	184	AFWWVMSCYQTMTSI	3.136	2.443	Cluster B
AaegGr57	300	NMVYNIFCSGFIIQL	2.522	2.443	Cluster B
AaegGr60	2	MCYRAVNIY	5.093	1.484	Cluster A
	336	LVHKAINCSTSSVVI	4.049	2.443	Cluster B
AaegGr62	53	WLNLLGNCISYLLVV	2.5	2.443	Cluster B
	337	VYKGITNCPSSAVKN	4.842	2.443	Cluster B
AaegGr63	6	MIGNICLFSSKPF	3.448	1.484	Cluster A
AaegGr64	72	STLGILQCVAACVGY	3.027	2.443	Cluster B
AaegGr66	333	LHQLSISCINQFRAL	2.495	2.443	Cluster B
AaegGr67	207	NLGFFVQCLDEIDEL	3.337	2.443	Cluster B
AaegGr69	110	KLLHFDQCYNAMINS	3.87	2.443	Cluster B
AaegGr73	104	ILSIFTVCDEKMRTM	3.56	2.443	Cluster B
	137	ACIVTLTCGGTLGGL	2.875	2.443	Cluster B
AaegGr76	107	IYWRQFMCNERIQQL	3.212	2.443	Cluster B
	341	LQRIGIVCLDSRLTE	2.788	2.443	Cluster B
	349	LDSRLTECIYGLSKV	2.549	2.443	Cluster B
	359	GLSKVVQCMQEETVM	3.06	2.443	Cluster B
	385	TTIIAASCSYLILLI	1.874	1.484	Cluster A
AaegGr77	291	FSIFSMFCIGALVSY	3.402	2.443	Cluster B
AaegGr79	221	LHVQILSCYMALINV	2.484	2.443	Cluster B
AaegGr80	7	MAILVACHYIMEKH	2.495	2.443	Cluster B

## Data Availability

All the relevant data have been provided within the manuscript.

## Supplementary Materials

The following supporting information can be downloaded at: https://www.mdpi.com/article/10.3390/ijms241512263/s1.

## References

[1] Andrew J., Bar A. (2013). Morphology and morphometry of *Aedes aegypti* adult mosquito. Annu. Res. Rev. Biol..

[2] Holeva-Eklund W.M., Young S.J., Will J., Busser N., Townsend J., Hepp C.M. (2022). Species Distribution Modeling of *Aedes aegypti* in Maricopa County, Arizona from 2014–2020. Front. Environ. Sci..

[3] Tabachnick W.J., Munstermann L.E., Powell J.R. (1979). Genetic distinctness of sympatric forms of *Aedes aegypti* in East Africa. Evolution.

[4] Barrera R. (1996). Competition and resistance to starvation in larvae of container-inhabiting *Aedes* mosquitoes. Ecol. Entomol..

[5] Zettel C., Kaufman P. (2009). Yellow fever mosquito *Aedes aegypti* (Linnaeus) (Insecta: Diptera: Culicidae). EDIS.

[6] Alberts B., Bray D., Hopkin K., Johnson A.D., Lewis J., Raff M., Roberts K., Walter P. (2015). Essential Cell Biology.

[7] Pask G.M., Ray A., Zufall F., Munger S.D. (2016). Insect Olfactory Receptors: An Interface between Chemistry and Biology. Chemosensory Transduction.

[8] Clyne P.J., Warr C.G., Carlson J.R. (2000). Candidate taste receptors in *Drosophila*. Science.

[9] McIver S., Siemicki R. (1978). Fine structure of tarsal sensilla of *Aedes aegypti* (L.) (Diptera: Culicidae). J. Morphol..

[10] McIver S.B. (1982). Sensilla of mosquitoes (Diptera: Culicidae). J. Med. Entomol..

[11] McIver S., Siemicki R. (1981). Innervation of cibarial sensilla of *Aedes aegypti* (L.) (Diptera: Culicidae). Int. J. Insect Morphol. Embryol..

[12] Lee R.M.K.W., Davies D.M. (1978). Cibarial sensilla of *Toxorhynchites* mosquitoes (Diptera: Culicidae). Int. J. Insect Morphol. Embryol..

[13] Robertson H.M., Warr C.G., Carlson J.R. (2003). Molecular evolution of the insect chemoreceptor gene superfamily in *Drosophila melanogaster*. Proc. Natl. Acad. Sci. USA.

[14] Matthews B.J., Dudchenko O., Kingan S.B., Koren S., Antoshechkin I., Crawford J.E., Glassford W.J., Herre M., Redmond S.N., Rose N.H. (2018). Improved reference genome of *Aedes aegypti* informs arbovirus vector control. Nature.

[15] Arensburger P., Megy K., Waterhouse R.M., Abrudan J., Amedeo P., Antelo B., Bartholomay L., Bidwell S., Caler E., Camara F. (2010). Sequencing of *Culex quinquefasciatus* establishes a platform for mosquito comparative genomics. Science.

[16] Sparks J.T., Vinyard B.T., Dickens J.C. (2013). Gustatory receptor expression in the labella and tarsi of *Aedes aegypti*. Insect Biochem. Mol. Biol..

[17] Nene V., Wortman J.R., Lawson D., Haas B., Kodira C., Tu Z.J., Loftus B., Xi Z., Megy K., Grabherr M. (2007). Genome sequence of *Aedes aegypti*, a major arbovirus vector. Science.

[18] Timoshevskiy V.A., Severson D.W., Debruyn B.S., Black W.C., Sharakhov I.V., Sharakhova M.V. (2013). An integrated linkage, chromosome, and genome map for the yellow fever mosquito *Aedes aegypti*. PLoS Neglected Trop. Dis..

[19] Dudchenko O., Batra S.S., Omer A.D., Nyquist S.K., Hoeger M., Durand N.C., Shamim M.S., Machol I., Lander E.S., Aiden A.P. (2017). De novo assembly of the *Aedes aegypti* genome using Hi-C yields chromosome-length scaffolds. Science.

[20] Ribeiro J.M.C., Hazzard J.M.H., Nussenzveig R.H., Champagne D.E., Walker F.A. (1993). Reversible binding of Nitric-Oxide by a salivary heme protein from a bloodsucking insect. Science.

[21] Walker F.A. (2005). Nitric oxide interaction with insect nitrophorins and thoughts on the electron configuration of the {FeNO} 6 complex. J. Inorg. Biochem..

[22] Damhus T., Hartshorn R., Hutton A. (2005). Nomenclature of inorganic chemistry: IUPAC recommendations 2005. Chem. Int..

[23] Davies S.-A. (2000). Nitric oxide signalling in insects. Insect Biochem. Mol. Biol..

[24] Nabi R.B.S., Tayade R., Hussain A., Kulkarni K.P., Imran Q.M., Mun B.-G., Yun B.-W. (2019). Nitric oxide regulates plant responses to drought, salinity, and heavy metal stress. Environ. Exp. Bot..

[25] Hussain A., Mun B.-G., Imran Q.M., Lee S.-U., Adamu T.A., Shahid M., Kim K.-M., Yun B.-W. (2016). Nitric oxide mediated transcriptome profiling reveals activation of multiple regulatory pathways in *Arabidopsis thaliana*. Front. Plant Sci..

[26] Hussain A., Yun B.-W., Kim J.H., Gupta K.J., Hyung N.-I., Loake G.J. (2019). Novel and conserved functions of S-nitrosoglutathione reductase in tomato. J. Exp. Bot..

[27] Imran Q.M., Falak N., Hussain A., Mun B.G., Sharma A., Lee S.U., Kim K.M., Yun B.W. (2016). Nitric Oxide Responsive Heavy Metal-Associated Gene AtHMAD1 Contributes to Development and Disease Resistance in *Arabidopsis thaliana*. Front. Plant Sci..

[28] Pande A., Mun B.-G., Lee D.-S., Khan M., Lee G.-M., Hussain A., Yun B.-W. (2021). NO network for plant–microbe communication underground: A review. Front. Plant Sci..

[29] Roberts Jr J.D., Lang P., Bigatello L.M., Vlahakes G.J., Zapol W.M. (1993). Inhaled nitric oxide in congenital heart disease. Circulation.

[30] Gusarov I., Gautier L., Smolentseva O., Shamovsky I., Eremina S., Mironov A., Nudler E. (2013). Bacterial nitric oxide extends the lifespan of *C. elegans*. Cell.

[31] Zhou J., Jia F., Shao S., Zhang H., Li G., Xia X., Zhou Y., Yu J., Shi K. (2015). Involvement of nitric oxide in the jasmonate-dependent basal defense against root-knot nematode in tomato plants. Front. Plant Sci..

[32] Sadekuzzaman M., Stanley D., Kim Y. (2018). Nitric oxide mediates insect cellular immunity via phospholipase A2 activation. J. Innate Immun..

[33] Müller U. (2012). The molecular signalling processes underlying olfactory learning and memory formation in honeybees. Apidologie.

[34] Trimmer B.A., Aprille J.R., Dudzinski D.M., Lagace C.J., Lewis S.M., Michel T., Qazi S., Zayas R.M. (2001). Nitric oxide and the control of firefly flashing. Science.

[35] Ohtsuki H., Yokoyama J., Ohba N., Ohmiya Y., Kawata M., Shelly T. (2014). Expression of the *NOS* gene and firefly flashing: A test of the nitric-oxide-mediated flash control model. J. Insect Sci..

[36] Stevenson P.A., Rillich J. (2015). Adding up the odds—Nitric oxide signaling underlies the decision to flee and post-conflict depression of aggression. Sci. Adv..

[37] Liu Y.-B. (2016). Nitric oxide fumigation for control of western flower thrips and its safety to postharvest quality of fresh fruit and vegetables. J. Asia-Pac. Entomol..

[38] Hussain A., Imran Q.M., Shahid M., Yun B.-W., Pratap Singh V., Singh S., Tripathi D.K., Romero-Puertas M.C., Sandalio L.M. (2022). Nitric oxide synthase in the plant kingdom. Nitric Oxide in Plant Biology.

[39] Hess D.T., Matsumoto A., Kim S.-O., Marshall H.E., Stamler J.S. (2005). Protein S-nitrosylation: Purview and parameters. Nat. Rev. Mol. Cell Biol..

[40] Elphick M.R., Green I.C., O’Shea M. (1993). Nitric oxide synthesis and action in an invertebrate brain. Brain Res..

[41] Bredt D.S., Snyder S.H. (1990). Isolation of nitric oxide synthetase, a calmodulin-requiring enzyme. Proc. Natl. Acad. Sci. USA.

[42] Saitou N., Nei M. (1987). The neighbor-joining method: A new method for reconstructing phylogenetic trees. Mol. Biol. Evol..

[43] Felsenstein J. (1985). Confidence Limits on Phylogenies: An Approach Using the Bootstrap. Evolution.

[44] Zuckerkandl E., Pauling L., Bryson V., Vogel H.J. (1965). Evolutionary divergence and convergence in proteins. Evolving Genes and Proteins.

[45] Kumar S., Stecher G., Tamura K. (2016). MEGA7: Molecular Evolutionary Genetics Analysis Version 7.0 for Bigger Datasets. Mol. Biol. Evol..

[46] Guruprasad K., Reddy B.V., Pandit M.W. (1990). Correlation between stability of a protein and its dipeptide composition: A novel approach for predicting in vivo stability of a protein from its primary sequence. Protein Eng. Des. Sel..

[47] Ikai A. (1980). Thermostability and aliphatic index of globular proteins. J. Biochem..

[48] Kyte J., Doolittle R.F. (1982). A simple method for displaying the hydropathic character of a protein. J. Mol. Biol..

[49] Voorrips R.E. (2002). MapChart: Software for the graphical presentation of linkage maps and QTLs. J. Hered..

[50] Hill C.A., Fox A.N., Pitts R.J., Kent L.B., Tan P.L., Chrystal M.A., Cravchik A., Collins F.H., Robertson H.M., Zwiebel L.J. (2002). G protein-coupled receptors in *Anopheles gambiae*. Science.

[51] Bailey T.L., Elkan C. Fitting a mixture model by expectation maximization to discover motifs in biopolymers. Proceedings of the International Conference on Intelligent Systems for Molecular Biology.

[52] Rockman M.V., Skrovanek S.S., Kruglyak L. (2010). Selection at linked sites shapes heritable phenotypic variation in *C. elegans*. Science.

[53] Campbell C.L., Dickson L.B., Lozano-Fuentes S., Juneja P., Jiggins F.M., Black W.C. (2017). Alternative patterns of sex chromosome differentiation in *Aedes aegypti* (L). BMC Genom..

[54] Iglesias M.J., Terrile M.C., Correa-Aragunde N., Colman S.L., Izquierdo-Álvarez A., Fiol D.F., París R., Sánchez-López N., Marina A., Calderón Villalobos L.I.A. (2018). Regulation of SCF(TIR1/AFBs) E3 ligase assembly by S-nitrosylation of Arabidopsis SKP1-like1 impacts on auxin signaling. Redox Biol..

[55] Terrile M.C., París R., Calderón-Villalobos L.I., Iglesias M.J., Lamattina L., Estelle M., Casalongué C.A. (2012). Nitric oxide influences auxin signaling through S-nitrosylation of the Arabidopsis TRANSPORT INHIBITOR RESPONSE 1 auxin receptor. Plant J. Cell Mol. Biol..

[56] Albertos P., Romero-Puertas M.C., Tatematsu K., Mateos I., Sánchez-Vicente I., Nambara E., Lorenzo O. (2015). S-nitrosylation triggers ABI5 degradation to promote seed germination and seedling growth. Nat. Commun..

[57] Tada Y., Spoel S.H., Pajerowska-Mukhtar K., Mou Z.L., Song J.Q., Wang C., Zuo J.R., Dong X.N. (2008). Plant immunity requires conformational changes of NPR1 via S-nitrosylation and thioredoxins. Science.

[58] Ayyar P.V. (2016). Uncovering the Role of S-Nitrosylation in Jasmonic Acid Signalling During the Plant Immune Response.

[59] Weichsel A., Maes E.M., Andersen J.F., Valenzuela J.G., Shokhireva T., Walker F.A., Montfort W.R. (2005). Heme-assisted S-nitrosation of a proximal thiolate in a nitric oxide transport protein. Proc. Natl. Acad. Sci. USA.

[60] Tamura K., Peterson D., Peterson N., Stecher G., Nei M., Kumar S. (2011). MEGA5: Molecular evolutionary genetics analysis using maximum likelihood, evolutionary distance, and maximum parsimony methods. Mol. Biol. Evol..

[61] Hall T.A. (1999). BioEdit: A User-Friendly Biological Sequence Alignment Editor and Analysis Program For Windows 95/98/NT.

[62] Waterhouse A., Bertoni M., Bienert S., Studer G., Tauriello G., Gumienny R., Heer F.T., de Beer T.A.P., Rempfer C., Bordoli L. (2018). SWISS-MODEL: Homology modelling of protein structures and complexes. Nucleic Acids Res..

[63] McGuffin L.J., Shuid A.N., Kempster R., Maghrabi A.H.A., Nealon J.O., Salehe B.R., Atkins J.D., Roche D.B. (2018). Accurate template-based modeling in CASP12 using the IntFOLD4-TS, ModFOLD6, and ReFOLD methods. Proteins.

[64] McGuffin L.J., Adiyaman R., Maghrabi A.H.A., Shuid A.N., Brackenridge D.A., Nealon J.O., Philomina L.S. (2019). IntFOLD: An integrated web resource for high performance protein structure and function prediction. Nucleic Acids Res..

[65] Roche D.B., Tetchner S.J., McGuffin L.J. (2011). FunFOLD: An improved automated method for the prediction of ligand binding residues using 3D models of proteins. BMC Bioinform..

[66] Xue Y., Liu Z.X., Gao X.J., Jin C.J., Wen L.P., Yao X.B., Ren J.A. (2010). GPS-SNO: Computational prediction of protein S-Nitrosylation sites with a modified GPS algorithm. PLoS ONE.

