# Supplementary figures and images for: In Silico Characterisation of the Aedes aegypti Gustatory Receptors
Source: Int J Mol Sci. 2023 Jul 31;24(15):12263. doi: 10.3390/ijms241512263 (PMC10419030; doi:10.3390/ijms241512263)

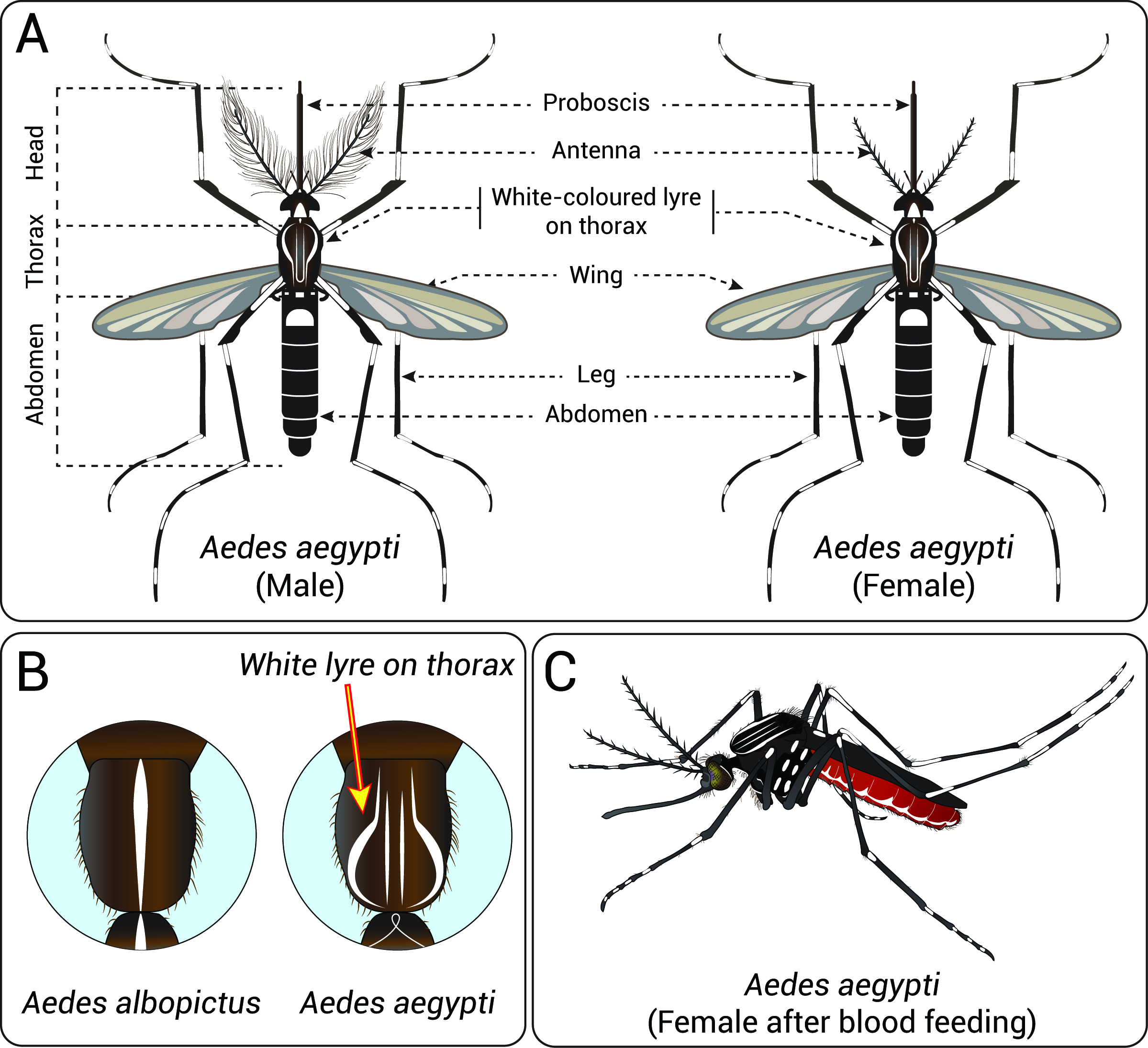

Supplement: Supplementary file 1 [file ijms-24-12263-s001.zip › ijms-2499327-supplementary.tif]
